# Highly Efficient Biosorption of Cationic Dyes via Biopolymeric Adsorbent-Material-Based Pectin Extract Polysaccharide and Carrageenan Grafted to Cellulosic Nonwoven Textile

**DOI:** 10.3390/polym16050585

**Published:** 2024-02-21

**Authors:** Yassine EL-Ghoul, Salman Alsamani

**Affiliations:** 1 Department of Chemistry, College of Science, Qassim University, Buraidah 51452, Saudi Arabia; 421100253@qu.edu.sa; 2 Textile Engineering Laboratory, University of Monastir, Monastir 5019, Tunisia

**Keywords:** polyelectrolyte multi-layers, suaeda fruticosa polysaccharide, extraction, carrageenan, cellulosic nonwoven, grafting, NMR, SEM, adsorption, isotherms

## Abstract

Water scarcity and contamination have emerged as critical global challenges, requiring the development of effective and sustainable solutions for the treatment of contaminated water. Recently, functionalized polymer biomaterials have garnered significant interest because of their potential for a wide range of water treatment applications. Accordingly, this paper highlights the design of a new adsorbent material based on a cellulosic nonwoven textile grafted with two extracted biopolymers. The layer-by-layer grafting technique was used for the polyelectrolyte multi-layer (PEM) biosorbent production. Firstly, we extracted a Suaeda fruticosa polysaccharide (SFP) and confirmed its pectin-like polysaccharide structure via SEC, NMR spectroscopy, and chemical composition analyses. Afterward, the grafting was designed via an alternating multi-deposition of layers of SFP polymer and carrageenan crosslinked with 1,2,3,4-butanetetracarboxylic acid (BTCA). FT-IR and SEM were used to characterize the chemical and morphological characteristics of the designed material. Chemical grafting via polyesterification reactions of the PEM biosorbent was confirmed through FT-IR analysis. SEM revealed the total filling of material microspaces with layers of grafted biopolymers and a rougher surface morphology. The assessment of the swelling behavior revealed a significant increase in the hydrophilicity of the produced adsorbent system, a required property for efficient sorption potential. The evaluation of the adsorption capabilities using the methylene blue (MB) as cationic dye was conducted in various experimental settings, changing factors such as the pH, time, temperature, and initial concentration of dye. For the untreated and grafted materials, the greatest adsorbed amounts of MB were 130.6 mg/g and 802.6 mg/g, respectively (pH = 4, T = 22 C, duration = 120 min, and dye concentration = 600 mg/L). The high adsorption performance, compared to other reported materials, was due to the presence of a large number of hydroxyl, sulfonate, and carboxylic functional groups in the biosorbent polymeric system. The adsorption process fitted well with the pseudo-first-order kinetic model and Langmuir/Temkin adsorption isotherms. This newly developed multi-layered biosorbent shows promise as an excellent adsorption resultant and cheap-cost/easy preparation alternative for treating industrial wastewater.

## 1. Introduction

The issue of organic-matter-induced water pollution is worldwide, with varying aspects and consequences based on the developed statuses of individual nations [1,2]. The concentrations of pollutants in products must be as low as possible. Prevention is therefore essential and is based on the following three aspects: the regulatory aspect involves setting standards; the health aspect includes, in particular, the technical control of installations; and finally, the scientific and technological aspect corresponds to the improvement of the pollution control processes [3,4].

Nevertheless, there is still a lot of work to be done, particularly with regard to textile effluents. These discharges are part of the worst-treated wastewater and are recognized for their physically powerful staining, increased pH variations, high chemical oxygen demand, and higher bio-toxicity to microorganisms [5]. Because it is less noticeable than direct pollution (odor, cloudiness), pollution from toxic organic waste is more subtle [6]. Aquatic fauna are not the only species concerned as the health of living things is gradually declining, their lives are being shortened, their progeny may be impacted by deformities, and their likelihood of developing cancer will rise [7,8,9]. By eating the meat of these living things, as well as fruits, vegetables, and other foods, we consume these same harmful pollutants without realizing it because we are part of the food chain.

Among the most harmful organic pollutants are dyes [10,11,12]. Large volumes of polluting dye discharges into wastewater are reportedly caused by the pulp, food coloring, paper, textile, and cosmetic industries. Living aquatic organisms are seriously endangered by these toxic and even carcinogenic colored wastewater pollutants [13,14,15].

This imposes the necessity and the vital urgency to find an adequate and effective solution to treat these highly toxic and harmful discharges. Different techniques have been proposed in this regard. The various biological treatments, filtration combined with coagulation, precipitation, coagulation/flocculation, ozonation, reverse osmosis, adsorption, ion exchange, and advanced electrochemical oxidation processes have been investigated for the treatment of textile dye discharges [16,17,18,19,20,21]. These technologies have revealed deficiencies and limitations given their high investment and operating costs. The processes based on reverse osmosis and ion exchange have proved to be more interesting given their ability to recover pollutant products during their elimination from the effluents [22,23,24]. However, these processes as well as those of advanced oxidation are not economically feasible due to their investment and relatively high operating cost.

Alternatively, the adsorption process using solid adsorbents has gained specific attention from researchers, revealing great effective potential to treat and remove organic pollutants in wastewater treatment. This technique is shown to be the most advantageous thanks to its straightforward design and cheap cost of investment. In this perspective, different adsorbents based on synthetic and natural polymers have been investigated for the elimination of dyes from contaminated waste [25,26,27,28]. Moreover, adsorbents based on textile materials are not often reported in the literature. A few studies have been conducted on the removal of cationic dyes via the use of certain pretreated synthetic textiles. In these studies, their chemical modification did not improve their low adsorption capacities due to their inert and hydrophobic character [29,30,31]. The functionalization of cellulosic textiles by some commercialized natural polymers such as k-carrageenan, alginate, and chitosan during our previous studies allowed effective biosorbents with excellent adsorption capacities [32].

In line with this emerging topic, the present research work suggests a new biosorbent material based on nonwoven cellulose grafted with a pectin polysaccharide extract and carrageenan polymer as two proposed potent natural adsorbents. The grafting is provided by the intermediate of a BTCA polycarboxylic acid as a crosslinking agent. The performance of the made adsorbent in eliminating cationic dyes (methylene blue in the current study) will be warranted by the different carboxylic acid, sulfate, and ester groups of the two grafted polyanions and the different hydroxyl functions presented in both the cellulosic support and the two biopolymers. The textile support material used is based on cellulose polymer, which is well known for its favorable hydrophilic properties [33,34,35,36]. The first polymer is a carrageenan biopolymer from edible red algae, a natural sulfated polysaccharide recognized for its gelling and biological properties [37,38]. The investigated pectin-like polysaccharide is extracted from Suaeda fruticosa leaves. The Suaeda fruticosa polysaccharide (SFP) has revealed, in the literature, various biological and bacteriological properties [39]. Thus, in the current paper, we will take advantage of both the different properties of the two crosslinked natural polysaccharides as well as the effectiveness of the new multi-layer grafting method to design a natural adsorbent that is bioactive and particularly capable of revealing excellent sorption capacity. In addition, such a textile material treated with robust and durable grafting offers the possibility and the potential for multiple reuses.

In our method, after the extraction and characterization of the SFP biopolymer, the latest natural polysaccharide and the carrageenan biopolymer are grafted on the cellulosic material in the presence of the BTCA polycarboxylic acid as a crosslinking agent. The different parameters of grafting, such as time and curing temperature, will be optimized. Then, different characterization analyses (FT-IR, SEM, TGA/DTA, and swelling capacity) will be carried out to evaluate the effectiveness of the designed PEM material and the pad–dry–cure grafting process. After that, the performance of the biosorbent will be assessed in various experimental settings, such as pH, temperature, time, and methylene blue concentration. The experimental data will finally be analyzed and modeled via the exploration of theoretical kinetic and isotherm equations.

## 2. Materials and Methods

### 2.1. Materials

A low-cost nonwoven textile based on cellulosic fibers was used as the base material for the prepared adsorbent. The material had a surface weight of 240 g/m^2^ and a thickness of 0.6 mm. The nonwoven material was produced via a calendaring thermal consolidation. 1,2,3,4-butanetetracarboxylic acid (BTCA), used as a crosslinking agent, and the iota-carrageenan (a tiny white powder) were acquired from Sigma-Aldrich (Taufkirchen, Germany). All chemicals and reagents employed for the extraction of the pectin-like polysaccharide or the grafting procedures were used without further purification. Methylene blue (MB, M.W = 319.85 g/mol, λ_max_ in water = 665 nm, chemical formula: C_16_H_18_ClN_3_S) as a reference of cationic dyes used as adsorbate was provided by the Central Drug House (India). Distilled water was used to prepare the MB solutions for the batch adsorption experiments.

### 2.2. Extraction of the SFP Polymer

A productive extraction process that our research team had previously published was examined [40] with slight adjustments. Three kilograms of Fresh Suaeda fruticosa leaves were chopped and repeatedly cleaned in distilled water. They were next ground and stored for three days at 40 °C in an oven after being dried. Following a depigmentation process in a soxhlet containing 95% ethanol, the resultant powder (2.4 kg) was extracted using citric acid using an ultrasonic bath (pH = 3.5, temperature = 70 °C, and duration = 3 days). The acidic extract was filtered, neutralized with NaOH solution, and then precipitated with 95% ethanol after the residue was removed. Before deproteination, the precipitate was dissolved in distilled water. Ultimately, using a Biobase Vacuum Freeze Dreyer in Shandong, China, the aqueous phase was dialyzed in water and lyophilized to provide 17% of SFP polymer.

### 2.3. SEC Analysis of the SFP Polymer

By using size exclusion chromatography analysis, the macromolecular properties of SFP were obtained. The viscometer detector, differential refractive index (RI), and multiangle light scattering were all included in the SEC device. For the analysis, DMF was used as a solvent. We leveraged an OmniSEC program for data analysis and extrapolation.

### 2.4. NMR Analysis

The ^1^H and ^13^C NMR spectra of the extracted SFP polymer were obtained via a Bruker Avance DRX 500 spectrometer (Bruker Instruments, Inc., Rheinstetten, Germany). Spectra were recorded in D_2_O (99.8 Atom% D). Chemical shifts in different spectra were expressed in ppm using tetramethylsilane as an internal standard. In each experiment, the number of scans was fixed according to the sample concentration.

### 2.5. Analysis of Carbohydrate Content in the SFP Extract

Considering galactose as a standard, the phenol-sulfuric acid technique was used to determine the carbohydrate content of SFP [41]. To assess the amount of uronic acid, the carbazole pathway was adopted, with galacturonic acid serving as a reference [42] (Bitter and Muir 1962). According to Lowry et al., the protein amount was identified using the Lowry method [43].

### 2.6. Designing of the Biosorbent Material

A pad–dry–cure grafting process was performed for the functionalization of different cellulosic materials. The samples were washed beforehand with distilled water at 40 °C. Then, they were passed into an impregnation solution containing 60 g/L of SFP, 40 g/L of BTCA (crosslinking agent), 60 g/L of carrageenan, 10 g/L of sodium hypophosphite (catalyst for the polyesterifiacation reaction), and 3 g/L of ammonium hydrogen phosphate. Distilled water was used as a solvent for the impregnation bath. Nonwoven samples were padded in the impregnation bath then roll-squeezed and dried at 100 ° C for 30 min. After that, the samples were thermofixed at variable times and curing temperatures. Finally, to eliminate the nonfixed polymers, the functionalized samples were washed with distilled water and dried for 40 min at 100 °C. To evaluate the grafting rate, the samples were weighed before and after functionalization. Equation (1) was used to calculate the grafting rate of the functionalized samples (expressed as % − Wt). For each value recorded, 10 replicates were investigated.

(1) $$\%-Wt=\frac{\left[mf-mi\right]}{mi}\times 100$$

Here, % − Wt is the grafting rate, and *m_i_* and *m_f_* represent the weights of samples taken before and after the grafting procedure.

### 2.7. Grafting Characterization Procedures

The analysis of the chemical grafting was evaluated via an infrared spectroscopy study. An FTIR spectrometer from Agilent Technologies equipped with an ATR system (attenuated total reflection) was used for the different assessments of the untreated and functionalized samples. The different spectra were measured from 4000 to 400 cm^−1^.

For an efficient adsorption behavior, the swelling capacity of the adsorbent is usually considered a crucial characteristic. Swelling measurements of virgin and functionalized cellulosic material were assessed via a gravimetric technique. Samples were first dried to determine the initial weight (*mi*). Samples were then impregnated for 2 days in distilled water. The time of impregnation was varied, and after wiping, the weight was measured for each duration of impregnation. Equation (2) was utilized to ascertain the rate of swelling:

(2) $$\%-SR=\frac{\left(mf-mi\right)}{mi}\times 100$$

Here, the weights of the dried and swollen samples are, respectively, *mi* and *mf*.

An FEI Quanta SEM microscope was explored for the assessment of the surface morphology of virgin and grafted PEM adsorbents. For the various measurements, the accelerating voltage was fixed at 5 KV. Varying magnifications were selected to identify the surface morphological appearance of different samples. To increase the samples’ conductivity, a thin layer of carbon was applied on their surfaces before SEM analysis.

### 2.8. Adsorption Batch Experiments

The different adsorption experiments were performed using a batch reactor where the MB dye was set in contact with the designed adsorbent and stirred vigorously at 140 rpm. Different condition parameters affecting the adsorption performance were studied, including the pH (varied from 3 to 9), time (ranged from 0 to 120 min.), concentration of the MB dye (varying from 25 to 1000 mg/L), and contact temperature (22, 40, and 60 °C). Following each experiment, the residual concentration was measured using a Shimadzu (UV-2600) UV-visible spectrophotometer.

The following Equation (3) was used to evaluate the amount, expressed as *q* (mg/g), of the MB dye adsorbed on the different samples grafted with the PEM polymeric system.

(3) $$q\;\left(mg/g\right)=\frac{C_{0}-C_{e}}{m}\times v$$

Here, *v* is the MB dye volume (L), *m* is the adsorbent weight (g), and *C*_0_ and *C_e_* are the original and residual concentrations (mg/L), respectively.

## 3. Results and Discussion

### 3.1. SEC Evaluation and Carbohydrate Content in the SFP Extract

Physicochemical evaluations of the naturally extracted polysaccharide isolated from the Suaeda fruticosa plant were determined using steric exclusion chromatography. The macromolecular properties and the carbohydrate amount of the polysaccharide are summarized in Table 1.

The polymer’s average molecular weight per number was around 131,000 g/mol. The isolated polysaccharide had a polydispersity value of 2.03. It was regarded as an extremely acceptable value, even for an extract of natural polymers. This value demonstrated the extracted polymer’s satisfactory homogeneity and the effectiveness of the extraction technique used.

The optimized natural extract’s primary components, as shown by the findings of the carbohydrate composition analysis, were 44.39% galacturonic acid and 55.61% neutral monosaccharides. This measured rate of galacturonic acid is somewhat lower than that from the same fruit grown in a different place (47.5%) [39] and greater than that found in other reported fruit pectins, such as in grapefruit peel (27.34%) and citrus peel (33.20%) [44]. These variable results are usually due to distinct fruit pectin origins, different cultivation regions, different plant parts investigated, or different extraction methods applied. Additionally, the isolated SFP did not contain any proteins according to the results of the Lowry technique. According to the literature, the natural polymer extract may be regarded as comprising pectin-like polysaccharides due to the higher determined quantity of Gal acid [39].

#### NMR Spectroscopy Analysis

**Figure 1:**
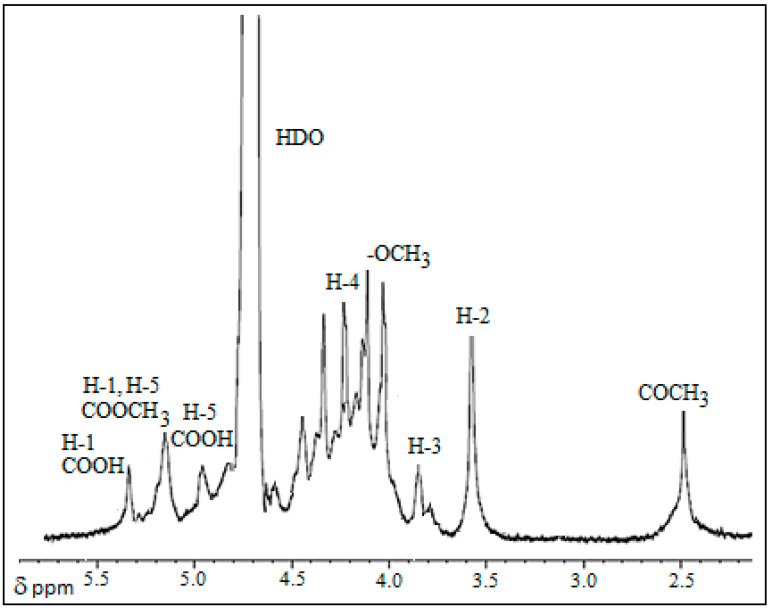
SFP extracted polymer—^1^H NMR spectrum.

To improve NMR spectra quality and to obtain more structure information, the SFP polymer was partially hydrolyzed before NMR analysis. Figure 1 shows the ^1^H spectrum of the extracted SFP. The different SFP signals were assigned according to the literature values. Two signals that appeared close to 5.18 and 4.94 were ascribed to the anomeric H-1 and H-5 protons from non-esterified GlA [45,46]. In addition, Tow overlapped signals appeared around d 5.16 and were assigned to the anomeric H-1 and H-5 from the esterified carboxyl groups in the galacturonic acid residues. The signal at 4.02 ppm showed the characteristics of protons of methyl ester that existed in (1,4)-linked GalA [47]. The signals at 3.66, 3.88, and 4.22 ppm were attributed to the H-2, H-3, and H-4 protons of (1,4)-linked GalA, respectively.

**Figure 2:**
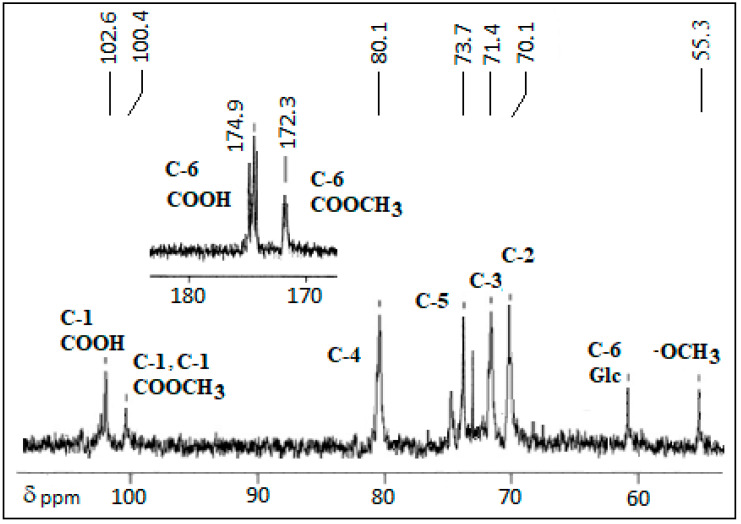
^13^C NMR spectrum of SFP extracted polymer.

The ^13^C NMR spectrum in Figure 2 showed two major signals that were assigned to the C-6 of the GalA carboxyl group at d 174.9 ppm (esterified) and d 172.3 ppm (non-esterified). Two anomeric signals appeared around d 102.1 and 101.0 ppm and were ascribed to the esterified and non-esterified GalA carboxyl units. Signals at 80.1 (C-4), 73.7 (C-5), 71.4 (C-3), and 70.1 (C-2) were attributed to the GalA carboxyl moieties [47]. The signal around 55.3 ppm was assigned to the O-CH3 ester group (O-methyl) linked to the C-6 [45]. Overall, ^1^H and ^13^C NMR analysis in turn confirmed the polysaccharide-like pectin structure of the SFP extracted polymer.

### 3.2. Preparation of the PEM Biopolymer Adsorbent

**Figure 3:**
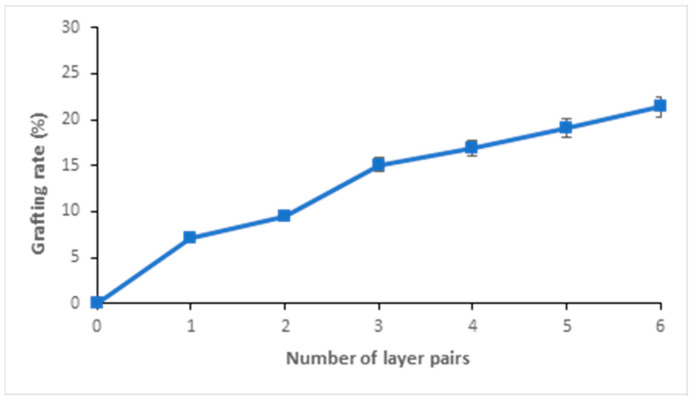
Grafting rate variation according to the number of layers grafted onto the biosorbent material.

The different biosorbent materials were designed via the layer-by-layer deposition technique. In this method, the materials obtained in the form of alternating layers of polymers contain one to six pairs of layers. The grafting rate variation is depicted in Figure 3 according to the number of pairings of successive layers of applied biopolymers. The weight gain increases progressively with the number of PEM layers grafted onto the cellulose biosorbent. We notice, from a functionalization of three pairs of polymers, the presence of a jump in the rate of grafting. Samples with three grafted pairs of layers will be selected for the characterization and adsorption studies.

### 3.3. Swelling Behavior

**Figure 4:**
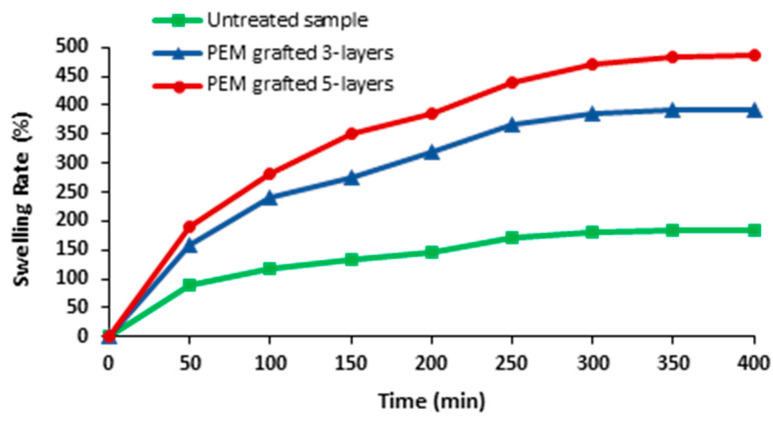
Variation in virgin and PEM biosorbent swelling capacities according to the number of grafted layers.

At varying periods of impregnation, swelling tests were conducted on functionalized PEM samples with varied pair layers and untreated cellulosic material. Results for the virgin cellulose material are shown in Figure 4, where a pseudo-plateau was reached upon 5 h, showing a maximum saturation of 184%. The swelling ratio increased gradually with time impregnation. Both of the functionalized PEM samples displayed a progressive rising trend. The sample that functionalized with three pairings had a swelling ratio more than twice as high as the untreated sample. The PEM material with five layers displayed a nearly three-fold swelling ratio, indicating a more hydrophilic capacitance. This resulted from the carrageenan biopolymer’s hydrophilic nature and the SFP extracted polysaccharide functionalizing the cellulosic material, both of which are renowned for their high hydrophilicity [48,49].

A superior hydrophilic composition exhibiting an increased water penetration capacity was attained through the functionalization process, employing natural hydrophilic biopolymers during the layer-by-layer deposition procedure. This is a necessary property to enhance adsorption capability.

### 3.4. FT-IR Analysis

**Figure 5:**
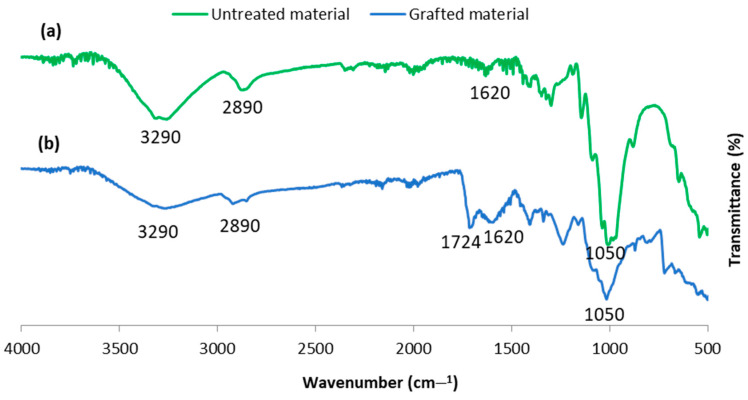
Spectra via FT-IR analysis of (**a**) untreated cellulose material and (**b**) grafted PEM adsorbent material.

FT-IR–ATR analysis was performed to identify the different functional groups that emerged during functionalization and to characterize the chemical grafting of the biosorbent material. This characterization was used to assess the PEM-grafted material as well as the virgin cellulose sample. Figure 5 displays the spectra of functionalized material with three polymeric layers and a cellulosic sample that had not been treated. The chemical grafting was demonstrated by various new peaks that developed in the PEM-grafted cellulosic material. The emergence of a peak with a center at 1724 cm^–1^ confirmed the polyesterification reaction between the hydroxyl functionalities of the SFP and carrageenan biological polymers and the carboxylic functions of the BTCA polycarboxylic acid. This is consistent with earlier research studies where FT-IR was employed to support the evidence of an esterification polymerization process using crosslinking compounds such as polycarboxylic acids to link cellulosic material to different polymers [25,50,51]. The grafted biosorbent material displays the various absorbances associated with the carrageenan sulfate groups, such as sulfate at 1214 cm^−1^, galactose-4-sulfate at 920 cm^−1^, and galactose-2-sulfate appearing at 834 cm^−1^ [40]. The SFP polymer’s symmetric vibration of the COO^−^ groups of the galacturonic acid was represented by the peak that was closed to 1310 cm^–1^ [52]. Additionally, a wider, more significant peak around 3290 cm^−1^ that was observed with treated material was assigned to the OH functions of the cellulose material, the SFP, and carrageenan biopolymers. In a nutshell, we were able to confirm the PEM biosorbent system’s chemical interconnection using FT-IR analysis, and the results revealed the effectiveness of the functionalizing chemical procedure using the layer-by-layer grafting technique.

### 3.5. SEM Analysis

**Figure 6:**
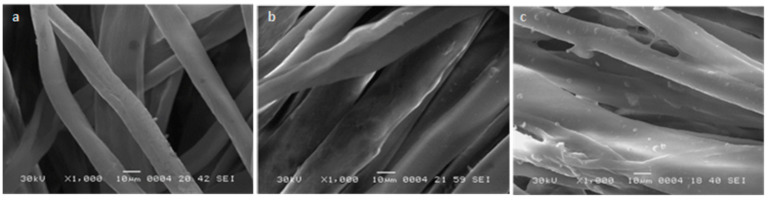
SEM analysis of virgin cellulose material (**a**) and grafted adsorbent designed at different layers: 3 layers (**b**) and 5 layers (**c**).

Figure 6 displayed micrographs of virgin and functionalized biosorbent materials with three- and five-layer pairs. After grafting, we observed a sizable surface change. The microspaces across the fibers were filled with the layers of the two grafted polymers and did not affect the surface porosity of the samples. When comparing the untreated sample to the grafted PEM, there was an increase in surface roughness. The significant change in surface morphology proved the permanence and efficacy of the grafting technique used.

### 3.6. Application to MB Dye Adsorption

**Figure 7:**
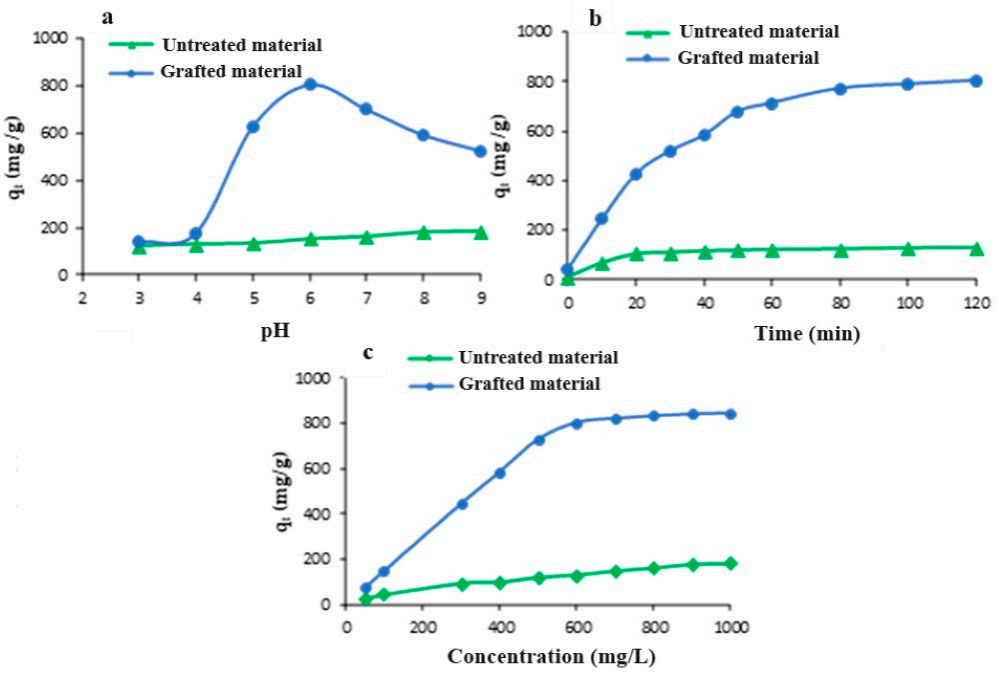
(**a**) Effect of pH (C_0_ = 600 mg/L, t = 120 min), (**b**) time (pH = 6, C_0_ = 600 mg/L, T = 22 °C), and (**c**) dye concentration (pH = 6, T = 22 °C, t = 120 min) on the adsorption of MB dye (samples are with 3 grafted pairs of layers).

In this study, the produced materials, both untreated and grafted, were used as MB dye adsorbents by modifying the pH, time, temperature, and dye concentration. The progress of the amount of adsorbed MB dye according to the pH variation is shown in Figure 7a. At pH = 6, the highest adsorbed amount was achieved. In actuality, poor adsorbed rates were caused by the positively charged methylene blue ions’ opposition to the adsorbent surface that is positively charged under highly acidic conditions. The adsorbent surface becomes negative at higher pH levels (pH ≈ 6), promoting an electrostatic interaction with the MB dye. The repulsive forces between dye molecules and the interface of the biosorbent could be the cause of the decreased amount of adsorbed dye under basic conditions.

The amount of time needed to reach equilibrium was found to be 120 min (Figure 7b). In the first 50 min, when more than 80% of the target was achieved, it can be seen that adsorption was quick. The fact that there are numerous adsorption sites available at the surface of the adsorbent during this initial stage may help explain this trend. Adsorption reached a constant state after this amount of time; it could be accounted for by the adsorption sites’ saturation. The highest amounts of methylene blue that could be adsorbed for untreated and grafted materials were 130.4 mg/g and 802.4 mg/g, respectively. The grafting of additional reactive functions (sulfonate and carboxylate groups) onto cellulose surfaces may account for this variation in sorption capacities.

**Figure 8:**
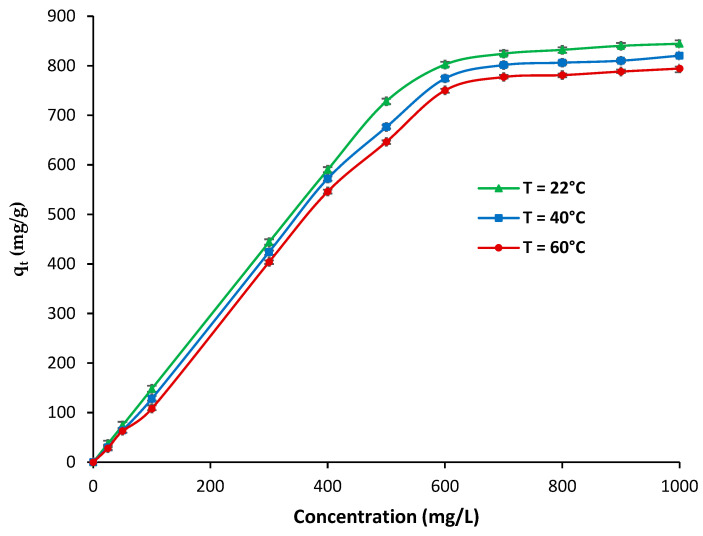
Effect of temperature on the MB dye adsorption (pH = 6, t = 120 min) (samples were with 3 grafted pairs of layers, and for each measurement, 3 replicates were made).

It was found that when the initial MB concentration increased, correspondingly, the amount of dye that was adsorbed increased. For the untreated samples, it reached 181.4 mg/g while, for the grafted materials it reached 802.6 mg/g (Figure 7c). Concerning functionalized samples, for instance, this adsorbed amount reduced from 844.5 mg/g at 22 °C to 794.5 mg/g at 60 °C as a result of the temperature variation (Figure 8). This demonstrated that the methylene blue adsorption in this instance was exothermic. Indeed, at higher temperatures, the MB dye may desorb from the samples.

Table 2 summarizes the adsorption performance of some recent natural polymeric adsorbents reported in the literature. The comparison with our studied adsorbent reveals the importance of the currently designed material as a potent biosorbent. The highest adsorption capacity reflects the adsorbent efficiency achieved and the effectiveness of the multi-layer process.

**Scheme 1:**
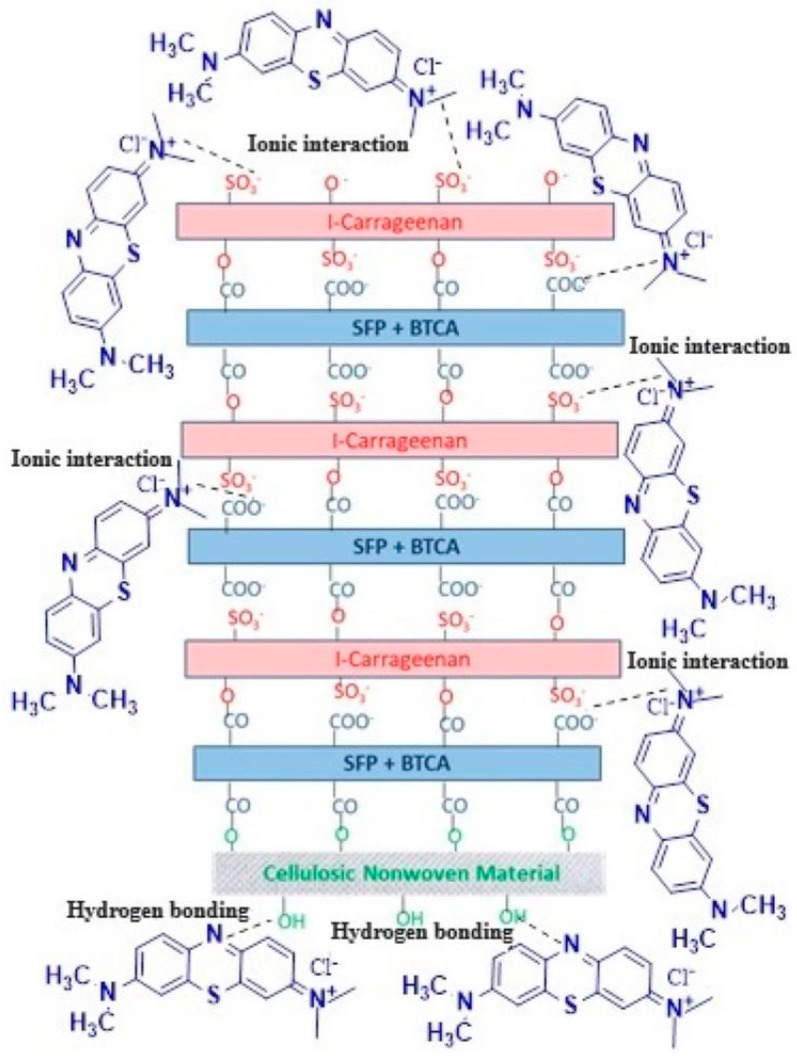
A schematic illustration of the different interactions between methylene blue dye and the textile nonwoven material grafted with the crosslinked polymers.

A schematic illustration of hydrogen bonds and ionic interactions between the PEM crosslinked to the surface of a cellulose textile material and MB dye molecules is shown in Scheme 1. Indeed, through the establishment of a hydrogen bond with the nitrogen atom of methylene blue, the free hydroxyl groups of cellulose nonwoven material might interact. However, through ionic interaction, the carboxylate groups of the SFP polymer (COO^−^) and BTCA crosslinking agent, as well as the sulfonate groups (SO_3_^−^ of carrageenan), may react with the N^+^ cations of the dye.

### 3.7. Kinetic Modeling

**Figure 9:**
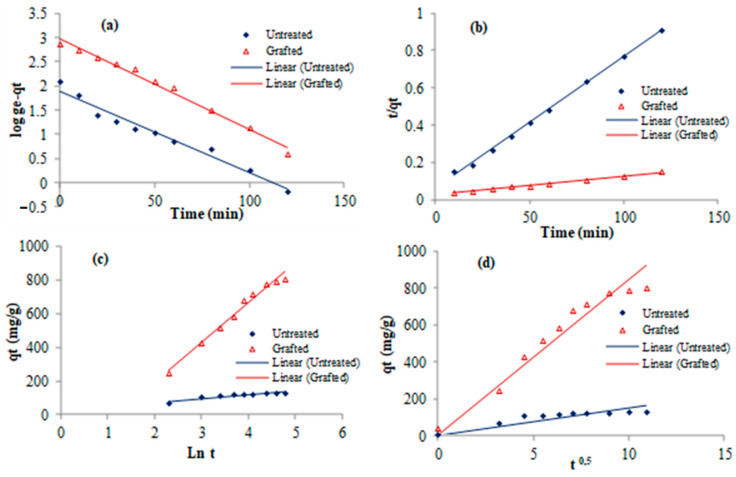
Adsorption kinetic data, mapped onto untreated and grafted biosorbent materials including (**a**) pseudo-first order, (**b**) pseudo-second order, (**c**) Elovich,, and (**d**) intraparticular diffusion models (samples are with 3 grafted pairs of layers).

To understand the equilibrium attraction between adsorbates and adsorbents, kinetic data were required. These might indicate whether the examined mechanism is physical, chemical, or involved in mass transfer. Here, the effectiveness of modeling kinetic data via Elovich and pseudo-first order, pseudo-second order, and intra-particular diffusion was evaluated. The outcomes are shown in Figure 9. In Table 3, the calculated kinetic parameters for the various equations are compiled. The calculated kinetic parameters and acquired curves suggested that the kinetic data might be well described by a pseudo-first-order equation (0.96 < R^2^). High correlation coefficients (0.99 < R^2^) were also achieved within the pseudo-second order. These findings demonstrated the complexity of the adsorption process, which could be classified as comprising both physical and chemical sorption modes [77]. The plots of the intraparticle diffusion model diverged from the starting point, indicating that it was not the only rate-controlling mechanism [78].

### 3.8. Isotherms and Thermodynamic Study

The equations [79] that follow are used to obtain the different thermodynamic parameters of the adsorption, which are the Gibbs free energy change (ΔG°), entropy change (ΔS°), and enthalpy change (ΔH°):

(4) ∆G°=RT×lnkd

(5) kd=qe×Ce

(6) $$lnkd=\frac{∆S{}^{\circ}}{R}-\frac{∆H{}^{\circ}}{RT}\;$$

ΔH° and ΔS° were obtained by plotting lnkd against 1/T, with the slope and intercept denoting the respective values.

**Figure 10:**
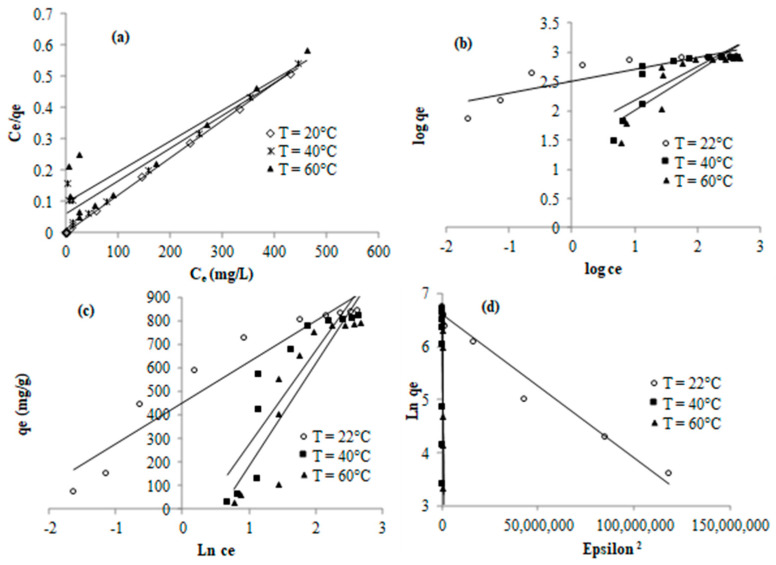
Models for isotherms data include (**a**) Langmuir, (**b**) Freundlich, (**c**) Temkin, and (**d**) Dubinin models.

The equations of Langmuir, Freundlich, Temkin, and Dubinin were used to assess the interaction between the grafted materials and the investigated adsorbate (Figure 10). Table 4 displays the derived parameters. In contrast to the other examined equations, the Langmuir equation showed a better fit with the experimental data (0.99 ≤ R^2^). This pattern implied a monolayer adsorption process and homogeneous sorption sites with comparable adsorption capabilities [80]. On the other hand, we observe that at 60 °C, the Temkin equation better fitted the adsorption data (0.91 ≤ R^2^) compared to the other models. This indicates that at relatively high temperatures (60 °C), the adsorption binding energy decreases with the increase in surface coverage [81]. Furthermore, at this temperature, we obtained a high value of the heat of sorption (the Temkin constant bt = 374.1 J mol^−1^), suggesting a chemical adsorption process [82].

The values of (1/n) exhibited surface heterogeneity or adsorption intensity. In fact, good adsorption may be displayed when the value of 1/n_F_ falls between 0.1 and 1.0 [83]. In our work, 1.72 ≤ n_F_ ≤ 2.41, which reveals that the methylene blue molecules adsorb so effectively to the surface of the grafted samples. The exothermic nature of the mechanism of adsorption was demonstrated by the drop in the adsorption energy constant values (B_T_), calculated using the Temkin model and a temperature rise. This is in line with the patterns shown in the temperature effect we mentioned previously.

The negative enthalpy value (ΔH° = −2.604 KJ/mol) suggests that the reaction between the RR198 reactive dye and the produced adsorbent is exothermic. The increase in adsorption capacity with temperature was supported by these data. The entropy change’s negative result (ΔS° = −56.103 J/mol) indicated that there was less disorder and randomness at the interface between the MB dye’s solid solution and the produced adsorbent. The computed free energy (ΔG° = 13.74 − 50.18 KJ/mol) showed positive values, indicating a non-spontaneous sorption mechanism.

## 4. Conclusions

In the current study, a method of extraction applied to a Suaeda fruticosa plant yielded a naturally occurring polysaccharide, and various physicochemical characterizations (SEC, carbohydrate content, and NMR study) validated its pectin-like polysaccharide structure. Then, a layer-by-layer grafting method was investigated to design a novel multi-layered polymeric biosorbent. Chemical and morphological characteristics were obtained for the produced cellulosic biosorbent material that was grafted with extracted pectin polysaccharide and carrageenan biopolymers. The stability of the multi-layer grafting and the chemical functionalization during the polyesterification process were confirmed through FT-IR/ATR and SEM analysis. Methylene blue dye was the adsorbate selected as the reference cationic dye for biosorption assessment on PEM grafting material. The impact of the various process parameters, including pH, temperature, time, and MB concentration, on the sorption equilibrium was studied, revealing their significant effect. The biosorbent material created revealed exceptional sorption capacities of MB, reaching 802.6 mg/g higher than several polymeric materials reported in the literature. The enhanced sorption of the dye was caused by the incorporation of various carboxylate and sulfonate moieties into the crosslinked carrageenan/SFP polymer grafted onto the cellulose biosorbent system. The relationship between the equations from the theory and the data from experiments indicated that the data on kinetics could be accounted for in both pseudo-first order and pseudo-second order models, suggesting that the adsorption process involved both chemical and physical interactions. In heterogeneous adsorption sites, the adsorption phenomena took place, revealing an exothermic and spontaneous process. Adsorption data were better suited for the Langmuir isotherm. They demonstrated that all adsorption sites on the biosorbent material were homogeneous and had the same adsorption efficiency and that the adsorption process was confined to a monolayer (due to the excellent fit with the Langmuir model), which was further confirmed by the non-adequacy obtained with the Frundlich model. We developed a straightforward and novel low-cost polymeric biosorbent material to remove one of the most harmful pollutants from the water discharges of the textile industry. It might be extended to investigate how this substance might be used to remove metals and pesticides among other contaminants.

## Figures and Tables

**Table 1:** Extracted SFP’s macromolecular properties and carbohydrate contents.

Extracted Product	M_n_ (g/mol)	M_w_ (g/mol)	*Đ* (M_w/_M_n_)	[ƞ] (mL/g)	Carbohydrate Content (%)		
SFP polysaccharide	131 × 10^3^	267 × 10^3^	2.03	289	Neutral sugar	Proteins	Gal Acid
					55.61	-	44.39

**Table 2:** An overview of the most recent studies on the adsorption of MB dye from industrial wastewater using natural polymeric absorbents.

Adsorbent	Adsorbate	q_t_ (mg/g)	Adsorption Efficiency (%)	Reference
k-carrageenan/alginate/cellulose	MB	522.4	98.6	[53]
Activated carbon from Date Press Cake	MB	613.8	83.3	[54]
Chitosan-epichlorohydrin/zeolite composite	MB	44.2	90	[55]
PAM-chitosan magnetic nanoparticles	MB	104.06	76.1	[56]
Chitosan/carboxymethyl cellulose capsules	MB	64.6	4.4	[57]
Chitosan/Zeolite composite	MB	19.23	84.85	[58]
Alginate/gelatin/graphene oxide composite	MB	322.6	-	[59]
Maize silk powder	MB	132.1	--	[60]
Prickly (peel) bark of cactus fruit	MB	222	-	[61]
Dead *Typha angustifolia* (L.) leaves	MB	106.75	89.83	[62]
watermelon rind	MB	200	99	[63]
Fe_3_O_4_–κ-carrageenan/chitosan	MB	123	-	[64]
Fe_3_O_4_@SiO_2_–κ-carrageenan	MB	530	-	[65]
Gelatin-based magnetic beads	MB	465	-	[66]
Carboxymethyl chitosan-modified magnetic-cored dendrimers	MB	20.85	-	[67]
Cactus pear seed cake	MB	260	56.48	[68]
Rattan (*Lacosperma secundiflorum*)	MB	359	96	[69]
Karanj fruit hulls	MB	239.4	94.4	[70]
Lignocellulosic Raspberry Leaves	MB	244.6	-	[71]
PAni@CTAB-Mt	MB	108.82	91.22	[72]
Chitosan/PAAM	MB	62.5	75	[73]
Alginate/PAA	MB	120	98	[73]
Cellulose Populus tremula seed fibers	MB	140.4	98	[74]
Alginate/polyethyleneimine	MB	400	99	[75]
Chitosan/carboxymethyl cellulose-GO	MB	405	82	[76]
SFP/carrageenan/cellulose PEM	MB	802.6	99	Current study

**Table 3:** Synopsis of the ungrafted and grafted samples’ kinetic data models.

Equations	Parameters		
		Untreated Material	Grafted Biosorbent
Pseudo-first order	K1 (1/min)	0.033	0.025
	q_e_ (mg/g)	79.305	962.948
	R^2^	0.969	0.988
Pseudo-second order	K_2_	0.000845	0.000036
	q	142.86	1000
	h	17.24	35.71
	R^2^	0.999	0.995
Elovich	α (mg/g/min)	72.447	78.046
	β (mg/g/min)	0.0042	0.044
	R^2^	0.982	0.857
Intra-particular- diffusion	K_1_ (mgg^−1^·min^1/2^)	15.168	84.507
	R^2^	0.956	0.989

**Table 4:** Different thermodynamic parameters and isotherm constants of adsorption.

T (°C)	Langmuir			Freundlich			Temkin			Dubinin			Thermodynamic		
	K_L_	q_L_	R^2^	K_F_	n_F_	R^2^	B_T_	A_T_	R^2^	q_m_	E	R^2^	∆G° (KJ/Mol)	∆H° (KJ/Mol)	∆S° (J/mol)
22	0.025	1000	0.999	321.07	2.044	0.772	438.3	0.07	0.821	740.40	2672.6	0.972	13.74	−2.604	−56.103
40	0.022	1000	0.932	40.07	2.411	0.642	400.8	0.73	0.80	773.29	707.1	0.851	31.50		
60	0.0093	1000	0.834	21.28	1.724	0.712	374.1	0.55	0.917	541.31	158.11	0.797	50.18		

## Data Availability

The data are contained within the article.
